# Enhanced Taste Recognition Following Subacute Treatment With The Dopamine D2/D3 Receptor Agonist Pramipexole in Healthy Volunteers

**DOI:** 10.1093/ijnp/pyac030

**Published:** 2022-05-23

**Authors:** Alexander Kaltenboeck, Don Chamith Halahakoon, Catherine J Harmer, Philip Cowen, Michael Browning

**Affiliations:** Department of Psychiatry, University of Oxford, Oxford, United Kingdom; Clinical Division of Social Psychiatry, Department of Psychiatry and Psychotherapy, Medical University of Vienna, Vienna, Austria; Department of Psychiatry, University of Oxford, Oxford, United Kingdom; Oxford Health NHS Foundation Trust, Warneford Hospital, Oxford, United Kingdom; Department of Psychiatry, University of Oxford, Oxford, United Kingdom; Oxford Health NHS Foundation Trust, Warneford Hospital, Oxford, United Kingdom; Department of Psychiatry, University of Oxford, Oxford, United Kingdom; Oxford Health NHS Foundation Trust, Warneford Hospital, Oxford, United Kingdom; Department of Psychiatry, University of Oxford, Oxford, United Kingdom; Oxford Health NHS Foundation Trust, Warneford Hospital, Oxford, United Kingdom

**Keywords:** Dopamine, gustation, pramipexole, taste, taste strip test

## Abstract

**Background:**

Patients with Parkinson’s disease (PD) show impaired performance in taste recognition tests, which suggests a possible dopaminergic influence on gustatory functioning. To experimentally test this hypothesis, we assessed whether pharmacological manipulation of dopaminergic signaling in healthy volunteers can affect performance in a standardized taste recognition test.

**Methods:**

Physically and mentally healthy volunteers (n = 40, age 18–43 years) were randomly allocated to treatment with either pramipexole or placebo using a double-blind, parallel-group design. After 12 to 15 days of treatment (dose titrated up from 0.25 mg/d of pramipexole salt to 1.0 mg/d), taste recognition performance was assessed using a standardized and validated assay (taste strip test). Additionally, visual analogue scale ratings of subjective pleasantness and disgustingness of taste samples were obtained.

**Results:**

Compared with the placebo group, participants receiving pramipexole showed significantly higher total recognition accuracy (median_pramipexole_ = 14.0, median_placebo_ = 13.0, U = 264.5, *P* = .04). This was driven by a higher sensitivity for taste in the pramipexole group. Exploratory analysis of pleasantness and disgustingness ratings of appetitive (sweet) vs aversive (bitter) stimuli suggested that pramipexole treatment was associated with overall blunted hedonic responses, but this effect did not survive the inclusion of nausea (a side effect of treatment) as a covariate in the analysis.

**Conclusions:**

Healthy volunteers who received subacute pramipexole treatment exhibited higher taste recognition performance compared with the placebo group. This finding is consistent with a proposed role of the dopaminergic system in gustatory functioning and could have important theoretical and clinical implications.

**Significance Statement**

Previous research suggests that patients with Parkinson’s disease show impairments in taste function. Whether this phenomenon can be attributed to altered dopaminergic neurotransmission is not clear to date. To experimentally test the influence of dopaminergic signaling activity on gustatory processing, the present study examined the effects of subacute treatment with the dopamine D2/D3 agonist pramipexole on performance in a taste recognition test in healthy volunteers. It was found that study participants who were treated with pramipexole performed significantly better in taste recognition compared with a control group treated with placebo. This was a global effect across different taste qualities rather than a taste-specific effect. Pramipexole-treated study participants also showed blunted hedonic evaluation of pleasant and unpleasant taste stimuli, but this effect could be attributed to nausea, a common side effect of pramipexole treatment.

## Introduction

Several studies have reported impaired taste recognition performance in patients with Parkinson’s disease (PD) (Lang et al., 2006; Moberg et al., 2007; Kim et al., 2011; Cecchini et al., 2014, 2015; Doty et al., 2015). These observations raise the possibility of a dopaminergic influence on gustatory functioning. However, in the aforementioned studies, patients were frequently receiving a range of medication when taste function was assessed, which means that any observed impairment could simply be a side effect of treatment. Additionally, central nervous system structures that contribute to the processing of gustatory information (e.g., nucleus tractus solitarius, operculum, insula, orbitofrontal cortex) could also be directly affected by neurodegenerative processes in PD (Shah et al., 2009; Cecchini et al., 2014; Doty et al., 2015). Furthermore, the neurochemistry of PD is complex, and a range of different neurotransmitter systems (e.g., serotonin, noradrenaline, acetylcholine)—all possibly also contributing to taste processing—is involved in the pathophysiology of the disease (Brichta et al., 2013). Thus, a direct role of dopaminergic signaling activity in gustatory functioning cannot be inferred from these observational studies.

The aim of this paper was to probe the putative causal role of dopamine in gustatory processing by assessing taste function after pharmacological manipulation of dopaminergic signaling activity. To this end, healthy volunteers were randomly allocated to subacute treatment with the dopamine agonist pramipexole or placebo and thereafter were assessed using a standardized taste assay. This approach allowed us to test the causal effects of dopaminergic activity on gustatory processing unconfounded by concurrent neuropathological processes or clinical symptoms.

Pramipexole is an orally active, non-ergoline dopamine agonist that shows selective activity at the D2 receptor subfamily, with a preferential affinity for the D3 receptor (Piercey, 1998; Tundo et al., 2019). It is an established treatment for PD and restless legs syndrome but has more recently also been suggested as a potential intervention for depressive disorders (Romeo et al., 2018; Tundo et al., 2019). A number of previous studies have used pramipexole to better understand the role of the dopaminergic system in different neurocognitive processes, including reinforcement and stimulus-response learning, impulsivity, and reward processing (Hamidovic et al., 2008; Pizzagalli et al., 2008; McCabe et al., 2013; Gallant et al., 2016; Martins et al., 2017). However, whether pramipexole can directly affect taste function has not, to our knowledge, previously been investigated.

Our working hypothesis for this paper was that, in healthy volunteers, pramipexole treatment would show opposite effects to those observed in PD, specifically, a global improvement in taste recognition. Because acute treatment with pramipexole has also been shown to blunt neural activity during the passive receipt of pleasant and unpleasant taste stimuli (McCabe et al., 2013)—including in brain areas thought to contribute to the hedonic experience of taste (Cecchini et al., 2015)—we additionally sought to explore whether pramipexole influenced subjective ratings of pleasantness and disgustingness in response to appetitive and aversive taste samples.

## METHODS

### Study Sample

Healthy volunteers (n = 40, 50% female) aged 18 to 43 years without a personal history of any major mental or physical disorder were recruited as part of a larger experimental medicine study that aimed to explore depression-relevant neurocognitive effects of subacute pramipexole treatment. In an initial screening visit, participants were confirmed to be mentally and physically healthy (based on a structured psychiatric interview (SCID-5 (First et al., 2015)) and a general medical interview), and basic demographic, physical, and psychological information was collected (also see Table 1).^1^ The trial was approved by the Oxford University research ethics committee, and all study participants gave written informed consent prior to inclusion.

**Table 1.** Basic Demographic, Physical, and Psychological Characteristics of the Study Sample (Means Plus SD in Parentheses)

	Pramipexole (n = 21; 10 male)	Placebo (n = 19; 10 male)	Between-group comparison (*t* test)
Age	22.5 (3.7)	24.5 (6.9)	*P* = .26
Body mass index	22.4 (2.6)	24.0 (2.9)	*P* = .07
Years in full-time education	16.8 (2.9)	17.5 (3.1)	*P* = .49
IQ estimate (Spot-the-Word Test)	108.3 (8.1)	111.9 (7.6)	*P* = .16
Neuroticism (Eysenck Personality Questionnaire)	4.2 (3.7)	4.3 (3.7)	*P* = .98
Psychoticism (Eysenck Personality Questionnaire)	2.5 (2.1)	2.8 (2.1)	*P* = .64
Extraversion (Eysenck Personality Questionnaire)	14.7 (4.5)	14.5 (3.7)	*P* = .89
Lie (Eysenck Personality Questionnaire)	9.5 (4.6)	7.5 (3.4)	*P* = .12
Trait anxiety (State-Trait Anxiety Inventory)	31.2 (9.1)	32.1 (9.1)	*P* = .77
Depression at inclusion (Beck Depression Inventory)	1.6 (1.7)	2.5 (4.0)	*P* = .39

N.B.: Uneven distribution of pramipexole and placebo treatment due to random replacement of participant dropouts.

### Intervention and Design

The study used a between-groups design with participants being randomly allocated to either pramipexole or placebo (lactose). Randomization was stratified by sex. Both treatments were administered in indistinguishable capsules. The daily dose of pramipexole started at 0.25 mg of pramipexole salt and was subsequently increased by 0.25 mg in a stepwise manner every 3 days until a target dose of 1.0 mg of pramipexole salt per day was reached. Participants took the target dose of 1.0 mg for at least 2 consecutive days before the taste assessment was conducted. Two participants dropped out of the study before the taste assessment because of subjectively experienced side effects and were subsequently replaced by other volunteers. Both participants who dropped out had received placebo treatment.

### Assessments

Taste recognition was assessed using a validated and standardized commercial test kit (Taste Strips, Burghart Messtechnik, Holm, Germany). Briefly, this assay consists of filter paper strips impregnated with 4 basic taste qualities (sweet, sour, salty, and bitter) at 4 different intensity levels (i.e., 16 samples in total). In addition, 3 strips without any taste (no-taste samples) were included. The filter paper strips were presented to participants in a pseudo-random order, with intensity levels increasing gradually, and no-taste samples being interleaved. In each trial, participants were asked to place the paper strip on their tongue, close their mouth, move the strip on their tongue, and then identify the taste. Participants were asked to correctly classify each sample as either sweet, sour, salty, bitter, or as having no taste. A total accuracy score was calculated for each participant as the number of samples correctly identified (e.g., sour presented and sample classified as sour). In addition, we also calculated a misidentification score, defined as the total number of trials where a participant incorrectly classified a sample with a taste quality (e.g., sour presented but sample classified as sweet). Furthermore, a no-taste identification score (no-taste sample presented and classified as such) and a non-identification score (taste sample presented but classified as having no taste) were calculated as well. To complement taste recognition measures, for each taste quality, we also calculated signal detection theory measures (based on Grier, 1971; see also Stanislaw and Todorov, 1999), which are able to determine sensitivity to a taste while controlling for response bias (for formulas, Supplementary Equation 1).

Additionally, participants were asked to rate each presented taste sample on 2 visual analogue scales (based on Arrondo et al., 2015) evaluating pleasantness (ranging from “very unpleasant” to “very pleasant”) and disgustingness (ranging from “not disgusting at all” to “extremely disgusting”).

Potential side effects of treatment (including sleeping problems, abnormal dreaming, headache, dizziness, somnolence, nausea, vomiting, constipation, fatigue, impulse control problems, hallucinations, and abnormal movements) were assessed using a simple side effect questionnaire that was administered before the administration of the taste test. Potential side effects noticed since beginning of treatment were rated by participants using a 4-level Likert scale ranging from “absent” to “severe.”

Blinding to treatment was checked by means of a forced-choice guess by the study participant and a researcher.

### Statistical Analysis

Data analysis was carried out in SPSS (version 25.0, IBM Corp, Armonk, NY). Taste recognition scores and hedonic ratings were compared between groups using Mann–Whitney U tests and mixed-design ANOVAs, respectively. For mixed ANOVAs, treatment group was always used as the between-participants factor and taste as the within-participant factor. To probe for a potential influence of nausea on the observed results, analyses were repeated with symptoms of nausea added as a covariate. Significant interactions in mixed-design ANOVAs were followed-up by a simple effects analysis using Bonferroni correction for multiple comparisons.

## RESULTS

### Taste Recognition

**Figure 1.**
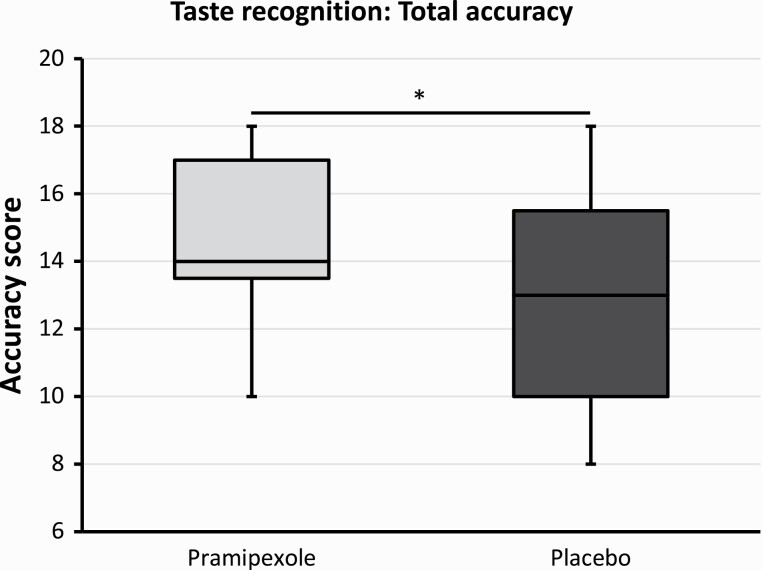
Boxplots comparing total taste recognition accuracy after subacute treatment with either pramipexole or placebo. Pramipexole group showing higher accuracy scores than the placebo group (*P* = .04).

One participant (allocated to the pramipexole group) had to be excluded from the analysis because of an error in the administration of the test (not all taste samples were presented). When comparing total accuracy scores (i.e., number of all correct classifications), there was a statistically significant difference between groups (U = 264.5, *P* = .04), with the pramipexole group showing higher accuracy compared with the placebo group (median_pramipexole_ = 14.0, median_placebo_ = 13.0) (also see Figure 1). This group difference was still present when the no-taste samples were excluded (U = 265.5, *P* = .03, median_pramipexole_ = 11.5, median_placebo_ = 10.0). Accuracy scores for no-taste samples alone did not show a significant group difference (U = 209.0, *P* = .61, median_pramipexole_ = 3.0, median_placebo_ = 3.0). Misidentification scores (i.e., number of incorrect classifications of a sample as a taste) did not differ significantly between groups (U = 188.5, *P* = .97, median_pramipexole_ = 3.0, median_placebo_ = 2.0). However, non-identification scores (i.e., number of incorrect classifications of a taste sample as having no taste) differed significantly between groups (U = 111.5, *P* = .03). Specifically, the pramipexole group exhibited fewer non-identifications than the placebo group (median_pramipexole_ = 1.0, median_placebo_ = 2.0).

To probe the potential influence of specific sample characteristics on recognition performance, we conducted group × taste mixed ANOVAs on taste accuracy, misidentifications, and non-identifications. This confirmed overall group differences (i.e., main effect of group) for accuracy (F_(1,37)_ = 5.5, *P* = .02) and non-identifications (F_(1,37)_ = 6.2, *P* = .02), but not misidentifications (F_(1,37)_ = 0.1, *P* = .81). However, there was no significant taste-specific group difference (i.e., no group × taste interaction) for any measure (accuracy: F_(3.1,113.3)_ = 1.4, *P* = .23; non-identifications: F_(3,111)_ = 0.7, *P* = .53; mis-identifications: F_(3,111)_ = 0.1, *P* = .95).

**Figure 2.**
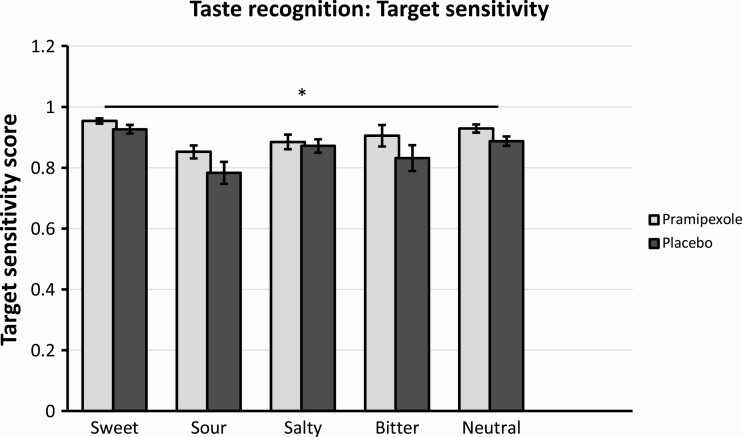
Target sensitivity scores for different taste qualities after subacute treatment with either pramipexole or placebo. Bars represent means, error bars represent standard errors. Main effect of treatment group, with pramipexole group showing higher target sensitivity independent of taste quality (*P* = .046).

Finally, we compared groups using signal detection theory measures (i.e., target sensitivity and response bias). In line with the results reported above, there was a significant main effect of group on target sensitivity (F_(1,37)_ = 4.2, *P* = .046) in the absence of a group × taste interaction (F_(2.4,89.9)_ = 0.7, *P* = .55). Compared with the placebo group, the pramipexole group showed significantly higher target sensitivity scores across taste qualities (also see Figure 2). There was no main effect of group (F_(1,37)_ = 0.2, *P* = .64) and no group × taste interaction (F_(2.7,99.2)_ = 0.95, *P* = .41) with regards to response bias scores.

### Pleasantness and Disgust Ratings of Taste Samples

To assess whether pramipexole influenced the hedonic experience of taste stimuli, we compared mean pleasantness and disgustingness ratings for sweet (i.e., appetitive) and bitter (i.e., aversive) taste samples between groups. As shown above, the placebo group classified significantly more taste samples incorrectly as having no taste, especially at the lower intensity levels (also see Supplementary Table 1), which confounds hedonic evaluations (i.e., one cannot meaningfully rate a taste that one does not perceive). Therefore, we compared only ratings for samples at the highest taste intensity level, where most participants identified taste samples correctly, and furthermore only included trials where a taste experience was reported. This analysis showed a significant group × taste interaction for both ratings, pleasantness (F_(1,34)_ = 6.5, *P* = .02) and disgustingness (F_(1,34)_ = 4.9, *P* = .03), but no main effect of group (pleasantness: F_(1,34)_ = 1.2, *P* = .29; disgustingness: F_(1,34)_ = 0.3, *P* = .62). Relative to the placebo group, the pramipexole group rated sweet samples as less pleasant and more disgusting and bitter samples as more pleasant and less disgusting. Comparing groups separately in terms of pleasantness and disgustingness ratings for sweet and bitter samples did not yield a statistically significant difference for either taste or measure (all Bonferroni-corrected *P* values > .05). Thus, the interaction was not driven by a group difference in a single taste quality.

### Can Nausea as a Side Effect Explain the Observed Results?

Nausea is a common side effect of pramipexole treatment and was the only side effect that was observed significantly more often in participants who received pramipexole (13 out of 21 participants, i.e., 61.9%) compared with placebo (1 out of 19, i.e., 5.3%) [Fisher’s exact test: *P < *.01; a detailed overview of all side effects observed in this study sample, can be found in Martens et al. (2021)]. Thus, it could be the case that the experience of nausea drove the observed group differences in gustatory processing. Indeed, an exploratory analysis showed a significant correlation between nausea and disgust ratings for sweet samples in the pramipexole group (*ρ* = 0.5, *P* = .03). We therefore reran the above analyses with nausea ratings added as a covariate. This led to no qualitative change in the results reported above for taste accuracy (F_(1,36)_ = 7.3, *P* = .01), non-identifications (F_(1,36)_ = 9.0, *P* < .01), and mis-identifications (F_(1,36)_ = 0.04, *P* = .85). However, when severity of nausea was included in the analysis for pleasantness and disgustingness ratings, the group × taste interaction reported above was no longer significant for either outcome measure (pleasantness: F_(1,33)_ = 2.1, *P* = .16; disgustingness: F_(1,33)_ = 2.2, *P* = .15).

### Blinding

Blinding in this study sample was not fully achieved. Pramipexole-treated participants correctly guessed their treatment allocation in 61.9% of cases and placebo-treated participants in 84.2% of cases (Fisher’s exact test: *P* < .01). The assessor correctly guessed treatment allocation in 71.4% of cases for pramipexole-treated participants and 84.2% of cases for placebo-treated participants (Fisher’s exact test: *P* < .01) (also see Martens et al., 2021).

## Discussion

In this paper, we used a healthy volunteer assay to study potential effects of subacute dopamine D2/D3 receptor agonism on gustatory functioning. We found that pramipexole-treated study participants exhibited enhanced taste recognition performance compared with placebo-treated volunteers. In addition, an exploratory analysis of hedonic ratings of different taste samples suggested that pramipexole-treatment was associated with blunted responses to both appetitive and aversive taste samples, but this pattern disappeared when we included symptoms of nausea as a covariate in the analysis.

As predicted, the observed effect of pramipexole was opposite to what has been described for PD patients (Lang et al., 2006; Moberg et al., 2007; Kim et al., 2011; Cecchini et al., 2014, 2015). This effect was seen across all taste qualities rather than being associated with a specific taste quality. The effect was also mainly driven by an increased sensitivity for detecting the presence of taste as evidenced by the fact that participants in the pramipexole group categorized significantly fewer samples incorrectly as having no taste. Importantly, no-taste recognition scores were comparable between groups, and, in a signal detection analysis, the pramipexole group also showed higher target sensitivity scores across all sample qualities. Therefore, enhanced taste recognition in the pramipexole group cannot be explained simply by an increased tendency to report a taste experience. The effect we observed also cannot be attributed to symptoms of nausea in the pramipexole group, because it was still observable when severity of nausea was included as a covariate in the analysis.

Taken together, the above results are in line with our initial hypothesis that gustatory processing is influenced by dopaminergic signaling activity. Therefore, decreased taste recognition performance in PD might be a direct result of impaired dopaminergic function. However, it is worth noting that a previous study that assessed taste performance in patients with early-stage PD both on and off dopamine-related medication (mostly carbidopa/levodopa) found no effect of dopaminergic drug treatment (Doty et al., 2015). Although this discrepancy could be explained by various methodological differences between that study and ours (e.g., a patient vs a healthy volunteer sample, different duration of treatment, etc.), it could also indicate that the effects we observed on taste recognition here are specific to pramipexole, for example, because of its specific affinity for the D3 receptor. Alternatively, because the neurochemistry of PD is complex and involves several other neurotransmitter systems than dopamine, it could be the case that taste recognition in PD patients is additionally influenced by non-dopaminergic pathology (Brichta et al., 2013). In line with this idea, Cecchini et al. (2019) recently demonstrated that chemosensory impairments in PD were associated with mild cognitive impairment, a symptom of PD that has also been linked to non-dopaminergic neurochemical abnormalities (Pasquini et al., 2021).

From a clinical perspective, our results suggest that pramipexole (and potentially other dopaminergic drugs) could represent a viable pharmacological treatment option for impaired gustatory function. This potential application deserves further investigation, especially given that medicine currently lacks effective therapies for taste disorders such as hypogeusia and ageusia (Kumbargere Nagraj et al., 2017). This seems particularly relevant in the context of the current COVID-19 pandemic, which will potentially leave some people suffering from long-term gustatory impairments (Vaira et al., 2020).

Interestingly, major depressive disorder is also associated with impaired chemosensory function, including reductions in both gustatory and olfactory sensitivity (Amsterdam et al., 1987; Deems et al., 1991; Berlin et al., 1998; Kohli et al., 2016). Recent work has suggested that pramipexole itself has antidepressant activity (Romeo et al., 2018; Tundo et al., 2019), raising the possibility that improved chemosensory function may be a mechanism by which dopaminergic agents, such as pramipexole, act to reduce symptoms of depression.

In addition to positive effects on taste recognition, we also observed that pramipexole-treated participants gave blunted subjective ratings of appetitive (sweet) and aversive (bitter) taste samples. Specifically, the pramipexole group showed lower pleasantness ratings for rewarding stimuli and higher pleasantness ratings for aversive stimuli, with a comparable pattern observed for ratings of disgustingness. Interestingly, this effect parallels previously reported observations at the neural level, whereby a single-dose of pramipexole reduced activity in the dorsal anterior cingulate cortex in response to both rewarding and aversive gustatory stimuli (McCabe et al., 2013). In this context, our finding is also noteworthy insofar as inhibitory effects of pramipexole on reward processing tend to be attributed to acute low-dose treatment, which is thought to primarily target presynaptic dopamine auto receptors (Pizzagalli et al., 2008; McCabe et al., 2013). Our results suggest that such an inhibitory effect can also be brought about by subacute treatment with pramipexole at a moderate dose. However, the pattern of blunted hedonic responses disappeared once nausea was included as a covariate in the analysis. Therefore, the group differences in hedonic ratings of appetitive and aversive taste stimuli could simply be due to increased nausea in the pramipexole group. Because these exploratory findings might be of clinical as well as theoretical relevance, they should be interrogated in closer detail in future trials.

Our study also has several strengths and limitations that require highlighting. To begin with, to the authors’ knowledge, this is the first assessment of gustatory function following subacute treatment with pramipexole. Because we utilized a healthy volunteer sample, as opposed to a patient sample, we were able to test potential effects of pramipexole on taste function unconfounded by neuropathology or changes in clinical symptoms. Compared with single-dose designs, commonly used for pharmacological manipulation in experimental settings, subacute drug treatment has higher clinical-ecological validity. This is especially important with pramipexole, because acute vs repeated dopaminergic manipulation might lead to differential neurochemical and neurocognitive effects (Newton et al., 2015; Martins et al., 2017). Another strength of this study is the taste test we used, which is a standardized and validated assessment system for gustatory function and is commercially available. This should make future replication efforts of our findings relatively straightforward.

There are also several limitations that need to be mentioned. First, we conducted a between-groups comparison with a moderately sized sample of participants. Although such a study design is cost-effective and practical to conduct, it might lack sensitivity to detect smaller effects (e.g., a taste-specific influence in addition to the global effect observed here). Second, as expected for a treatment with notable side effects, blinding was not fully achieved. Therefore, theoretically, treatment expectations could have confounded some of the observed results (however, one might be skeptical whether healthy volunteers are likely to hold strong expectations about pramipexole’s effect on gustatory processing). Third, by relying on a single, whole-mouth taste test and using only 1 specific target dose (i.e., 1.0 mg/d of pramipexole salt), the study was limited in terms of its methodological generalizability. Future investigations should employ alternative gustatory assessments (e.g., regional taste assessment and electrogustometry, gustatory evoked potentials, neuroimaging during taste recognition) to better understand the effects of dopaminergic manipulation on different levels of the gustatory processing hierarchy (e.g., tongue, cranial nerves, cortical areas, etc.). In addition, future studies should also try to establish a dose response curve for pramipexole’s effect on taste recognition. Fourth, the age range of our study participants was between 18 and 43 years, whereas PD typically manifests at around 50 to 60 years of age. Thus, our sample’s physiological, psychological, and social characteristics might not fully parallel those of PD patients. Finally, the investigation discussed here only utilized a healthy volunteer sample. As a next step, our findings should be independently replicated in clinical populations (e.g., in patients suffering from hypogeusia/ageusia or in patients with major depression) to explore the potential use of pramipexole (and other dopaminergic drugs) as a pharmacological treatment for impaired chemosensory function.

## CONCLUSION

In conclusion, this study found that subacute treatment with the dopamine D2/D3 receptor agonist pramipexole was associated with enhanced taste recognition performance in healthy volunteers. In addition, pramipexole-treated participants also showed blunted subjective evaluation of both appetitive and aversive taste stimuli, but this pattern was linked to the experience of nausea as a side effect. Taken together, our findings indicate a direct influence of dopaminergic signaling on gustatory processing, which could have several important theoretical and clinical implications.

## Supplementary Material

pyac030_suppl_Supplementary_Material

## Footnotes

1 Since treatment allocation was conducted in a randomized way, no specific further screening for conditions potentially influencing taste performance was conducted.

## Interest Statement

Catherine Harmer reports receiving grants from Johnson & Johnson, UCB, and Sunovion and personal fees from P1vital, Sage Therapeutics, Johnson and Johnson, Pfizer, Zogenix, Compass Pathways, and Lundbeck (outside this work). Michael Browning was supported by a MRC Clinician Scientist Fellowship (MR/N008103/1), has received travel expenses from Lundbeck for attending conferences, owns shares in P1vital Products Ltd., and acted as a consultant for Johnson & Johnson and CHDR. All other authors report no conflicts of interest.
