# Supplementary material for: Enhanced Taste Recognition Following Subacute Treatment With The Dopamine D2/D3 Receptor Agonist Pramipexole in Healthy Volunteers
Source: Int J Neuropsychopharmacol. 2022 May 23;25(9):720–6. doi: 10.1093/ijnp/pyac030 (PMC9515131; doi:10.1093/ijnp/pyac030)
Supplement: pyac030_suppl_Supplementary_Material [file pyac030_suppl_supplementary_material.pdf]

Supplements to Kaltenboeck et al.: "Enhanced taste recognition following subacute treatment with the dopamine D2/D3 receptor agonist pramipexole in healthy volunteers"

Supplementary Table 1: Overview of correct classifications per taste, intensity, and treatment group. Taste intensities range from I to IV with IV the highest taste intensity.

Taste	Intensity	Correct Placebo	Correct Pramipexole
Sweet	IV	100.0%	100.0%
Sweet	III	94.7%	95.0%
Sweet	II	68.4%	90.0%
Sweet	I	57.9%	75.0%
Sour	IV	94.7%	95.0%
Sour	III	63.2%	80.0%
Sour	II	15.8%	50.0%
Sour	I	0.0%	15.0%
Salty	IV	89.5%	90.0%
Salty	III	57.9%	70.0%
Salty	II	78.9%	70.0%
Salty	I	42.1%	55.0%
Bitter	IV	73.7%	90.0%
Bitter	III	73.7%	90.0%
Bitter	II	63.2%	75.0%
Bitter	I	31.6%	70.0%
Neutral Sample 1	-	89.5%	95.0%
Neutral Sample 2	-	78.9%	85.0%
Neutral Sample 3	-	100.0%	90.0%

Supplementary Equation 1: Formulas for calculating target sensitivity and response bias where H denotes Hit rate and F denotes false alarm rate (Grier, 1971; Stanislaw & Todorov, 1999).

$$Target\ sensitivity = \begin{cases} 0.5 + \dfrac{(H-F)(1+H-F)}{4H(1-F)} & \text{if } H \geq F \\ 0.5 - \dfrac{(F-H)(1+F-H)}{4F(1-H)} & \text{if } H < F \end{cases}$$

$$Response\ bias = \begin{cases} \dfrac{H(1-H)-F(1-F)}{H(1-H)+F(1-F)} & \text{if } H \geq F \\ \dfrac{F(1-F)-H(1-H)}{F(1-F)+H(1-H)} & \text{if } H < F \end{cases}$$
